# Influence of solidification structure on austenite to martensite transformation in additively manufactured hot-work tool steels

**DOI:** 10.1016/j.actamat.2021.117044

**Published:** 2021-08

**Authors:** Chia-Ying Chou, Niklas Holländer Pettersson, A. Durga, Fan Zhang, Christos Oikonomou, Annika Borgenstam, Joakim Odqvist, Greta Lindwall

**Affiliations:** a KTH Royal Institute of Technology, Brinellvägen 23, SE-10044 Stockholm, Sweden; b Material Measurement Laboratory, National Institute of Standards and Technology, 100 Bureau Drive, Gaithersburg, MD 20899, USA; c Uddeholms AB Uvedsvägen, SE-68333 Hagfors, Sweden

## Abstract

The microstructure of a hot-work tool steel additively manufactured using laser powder-bed fusion (L-PBF), and its response to post heat treatment, is studied in detail by microstructure characterization and computational thermodynamics and kinetics. The high solidification and cooling rates during the L-PBF process lead to suppression of *δ*-ferrite and instead solidification of an austenite phase directly containing a cellular substructure where the alloying elements have segregated to the inter-cellular regions and where solidification carbides have formed in the cell junctions. The austenite is then partly decomposed into martensite at lower temperatures. The micro-segregation can be predicted by reducing the complex solidification behavior to a diffusion problem in one dimension enabling detailed comparisons with the measured segregation profiles quantified at a nanometer scale. Martensite start temperature (M_s_) calculations along the spatially varying composition show that the M_s_ temperature decreases in the inter-cellular regions where austenite is observed. The network of austenite in the as-built microstructure can be understood from the combined influence of the composition dependence of the M_s_ temperature in relation to the build plate temperature and the mechanical stabilization of the small-sized austenite regions. This work demonstrates the power of computational tools based on computational thermodynamics and kinetics for designing tool steels for additive manufacturing by predictions of the steel’s response to the L-PBF process and post heat treatments.

## 1. Introduction

The processing of high-strength martensitic steels by laser-powder bed fusion (L-PBF) is of great interest for the tooling industry as it offers innovative solutions both in terms of tool and alloy design. Complex die shapes with conformal cooling channels are, for example, possible which may increase the die lifetime significantly by allowing for improved cooling efficiency and reduced heat checking effects. In addition to enlarging the geometrical design space, additive manufacturing (AM) via L-PBF yields manufacturing conditions significantly different from conventional manufacturing methods, enabling unique microstructures and new post-processing routes.

Martensitic, medium carbon steels alloyed with chromium, vanadium and molybdenum are common in hot-work tooling applications such as high-pressure aluminum die-casting as they offer a combination of resistance to heat checking, gross cracking, hot wear and plastic deformation [1]. Conventional hot-work tool steels may be manufactured using electro-slag-remelting followed by homogenization and thermo-mechanical treatments to break up the coarse solidification structure. The final microstructure is obtained by a hardening treatment consisting of austenitization followed by quenching to form martensite. The martensite is then tempered at relatively high temperatures, ~600 °C, so that carbide forming elements can diffuse and form fine secondary carbides. The precipitates contribute to secondary hardening of the material and their ability to not coarsen during service determines the temper resistance of the tool. The austenitization prior to tempering is performed at a temperature where a small fraction of primary carbides is stable in the austenitic matrix. The presence of these carbides limits the growth of the austenitic grains, but they also consume carbide forming alloying elements which are not any longer available for the secondary carbide precipitation during tempering.

The nominal compositions of hot-work tool steels are therefore a compromise between limiting the fraction of primary carbides forming at the austenitization temperature while maximizing the fraction of secondary carbides precipitating during tempering and in service.

In the case of L-PBF, the material is rapidly melted, solidified, and further quenched, at extremely high cooling rates, to temperatures below the martensite start (M_s_) temperature. This produces a martensitic microstructure with a fine-scaled solidification substructure [2–5] due to elemental micro-segregation. The question arises as to how to predict this solidification substructure and how it influences the transformation of austenite to martensite and the microstructure evolution during the austenitization. In addition, during L-PBF processing of such high-alloyed steels, the build plate is typically held at elevated temperatures in order to minimize the residual stresses in the manufactured material [3–6]. This, in combination with the complex thermal history, makes it crucial to be able to predict the variation in the M_s_ temperature in relation to the build plate temperature and in that way control the level of retained austenite in the built components. The M_s_ temperature can be predicted based on the chemical composition [7,8], however, the spatial variation of the M_s_ temperature in the micro-segregated microstructures of additively manufactured steels has not yet been calculated. To understand the implications of chemical heterogeneities and be able to control them are crucial for all manufacturing processes. In the case of conventionally manufactured armour and pressure-vessel steels, for example, banding occurs due to elemental segregation during casting [9,10]. This, in turn, may cause spatially different microstructure evolution upon heat treatments [9] or in the heat affected zones during welding [10], leading to mechanical heterogeneities and consequently inconsistent mechanical properties. Elemental segregation, when controlled, can also be advantageous. Raabe et al. [11], for example, explored a “segregation engineering” approach to manipulate the structure and composition of grain boundaries in a maraging steel via segregation and phase transformation. They showed that nano-sized austenite reversion layers can be stimulated to form at segregation decorated martensite lath boundaries which opens up for design of ductile martensite.

To explore the possibility to make use of the micro-segregation occurring during L-PBF and to optimize the mechanical properties of additively manufactured martensitic steels both to improve processability as well as component performance, a better understanding of the formation of the as-built microstructure and its response to post-heat treatments is needed. This includes adaptation of computational tools to be used in an integrated computational materials engineering (ICME) based materials design approach. In this work, the as-built microstructure of a vanadium and molybdenum alloyed hot-work tool steel manufactured by L-PBF is studied in detail by experimental microstructure characterization combining scanning and transmission electron microscopy (SEM and TEM) and synchrotron high-energy X-ray diffraction (HEXRD). Computational thermodynamics and kinetics are applied to predict the microstructure evolution during L-PBF, including the influence of solidification substructure on the variation of the M_s_ temperature. The primary carbides expected to form during austenitization heat treatment are also evaluated. The validity of the computational approach and the predicted microstructure evolution and heat treatment response of the L-PBF processed hot-work tool steel are discussed in detail.

## 2. Experimental methods

A hot-work tool steel was additively manufactured using a L-PBF system by SLM Solutions, SLM^®^ 280HL^1^. The feedstock material was nitrogen gas atomized powder, sieved at 20-50 μm size fraction. The nominal chemical composition is listed in Table 1. Cylinder shaped specimens were built using a laser power of 260 W, scan speed of 900 mm/s, layer thickness of 0.03 mm and hatch distance of 0.12 mm resulting in a volumetric energy density of about 80 J/mm^3^ and parts with >99.9 % density. The build plate temperature was kept at 200 °C during the whole process. The cylinder parts were oriented 30° to the building direction. Samples were taken from the cylindrical samples for microstructure characterizations. To allow for comparisons between the as-built microstructure and the austenitized microstructure, samples were also heat treated for 1 h at 1010 °C followed by water quenching.

The as-built and the austenitized samples were sectioned to observe the microstructure perpendicular and parallel to the building direction. The samples for SEM were ground and polished to 1 *μ*m-sized diamond paste finish, and the final polishing was done using colloidal alumina and silica suspensions. The microstructure was investigated by electron channeling contrast imaging (ECCI) and electron backscattered diffraction (EBSD) using a Jeol 7800F field-emission gun microscope equipped with the Bruker e-FlashHD EBSD system. Imaging was performed at 10 kV, while 15 kV was used for the EBSD analyses. Post processing of EBSD was done using MTEX crystallographic toolbox v5.1.1 [12] and the prior austenite grain structure was reconstructed according to the procedure proposed by Nyyssönen et al. [13].

The solidification structure and micro-segregation pattern were mapped by energy dispersive X-ray spectroscopy (EDS) at high spatial resolution using scanning transmission electron microscopy (STEM). Thin foils were prepared by electrolytic polishing using a twin-jet polisher and a solution of 15% perchloric acid in methanol held at −20 °C. The thin foils were argon ion beam polished prior to analyses. Carbon extraction replica samples were also prepared to allow for analysis of the precipitates without interaction of the matrix. Samples were slightly etched in 2% nital solution and coated by a 15 nm-thick carbon film using Gatan precision etching and coating system; the carbon film was etched free from the surface in a 5:1:5 solution of HCl, HNO_3_ and H_2_ O. The etched-off pieces of carbon were washed in ethanol, unfolded in distilled H_2_O before being transferred to Cu grids. The TEM analyses were performed at 200 kV using a JEOL 2100F field emission gun TEM equipped with a windowless X-Max^N^ detector for EDS.

The amount of austenite was measured by HEXRD in a transmission mode at beamline 11-ID-B of Advanced Photon Source, Argonne National Laboratory. The monochromatic X-ray wavelength was 0.2113 Å, which corresponds to an X-ray energy of 58.59 keV. Each sample was measured 200 times with individual exposure of 0.1 s to achieve high signal-to-noise and to improve statistics. HEXRD samples had a thickness of 1 mm, ensuring bulk crystal structures are measured, in contrast to surface crystal structures acquired using in-house XRD setups. Each surface was ground and polished to 1 μm-sized diamond finish at low load to avoid possible deformation-induced martensite. A 2D detector was used to eliminate the texture effect from the sample.

## 3. Computational methods

To predict the extent of micro-segregation during solidification, both the Scheil module and the diffusion module (DICTRA) within the Thermo-Calc Software package [14] were applied in the present work.

The Scheil-Gulliver model for solidification [15,16] has commonly been applied as the first approximation to predict the solute redistribution during AM [17–20]. The model can be used for multicomponent systems when coupled to a Calphad thermodynamic description of the system. The model assumes perfect mixing in the liquid phase, no diffusion in the solid phase and that local equilibrium holds at the liquid/solid interface. Thus, the Scheil-Gulliver model does not account for back-diffusion of elements or back-transformation of phases. In steel systems, back-diffusion of interstitial elements such as C and N can have a significant influence on the solidification process. In the Scheil-Gulliver model implemented in Thermo-Calc, this has been handled by allowing for elements to be entered as so-called *fast diffuser* which means that their diffusion is assumed to be infinite also in the solid phase. In this work, C and N were entered as *fast diffusers* for the Scheil-Gulliver simulation.

To fully account for the diffusion of all elements during solidification, DICTRA was applied. DICTRA solves the liquid/solid phase transformation in one dimension. A sharp interphase boundary is assumed and that local equilibrium holds at the interface. To perform a DICTRA solidification calculation, information about the size of the computational domain and the temperature as a function of time are needed. In this work, experimental characterization of the as-built solidification structure was used to determine the computational domain size and the temperature was assumed be linearly decreasing with time. Calculations were performed for a computational domain size of 300 nm based on the cellular solidification structure with a radius of about 300 nm and for cooling rates in the range typically suggested for the L-PBF process, i.e., 10^4^−10^6^ K/s [21,22].

For the DICTRA solidification calculations, only the liquid to austenite (fcc) transformation was simulated, i.e., the formation of β-ferrite (bcc) was not included. Also, for the Scheil simulations, *δ*-ferrite was suspended. According to the thermodynamics of this steel, *δ*-ferrite is the solid phase stable at the highest temperature and would be the first solid phase to form during solidification from a thermodynamic point of view. However, in several studies of phase selection in laser remelting [23] and welding [23,24], it has been shown that, for certain steel systems when a dendritic structure forms at high solidification velocities, austenite may have a higher dendrite tip temperature, and therefore a higher driving force to form compared to *δ*-ferrite.

In this work, the dendrite growth kinetics was studied using the Kurz-Giovanola-Trivedi (KGT) model [25]. The KGT model was further developed by Rappaz et al. [26] for multicomponent systems and was used by Fukumoto et al. [23] and Babu et al. [24] to study the dendrite growth kinetics of *δ*-ferrite and austenite under rapid solidification conditions. The model is based on the marginal stability criterion derived from a linear stability analysis of a planar solid-liquid interface undergoing directional solidification. The smallest unstable wavelength of perturbation introduced at the planar front is taken as the dendrite tip radius (R), which then shifts the interface temperature (or the dendrite tip temperature) due to the curvature undercooling contribution. Further, the effect of rapid solidification has been taken into account using the model by Aziz [27], which was further extended by Ahmad et al. [28]. The following equations are solved iteratively [24], see Appendix. Supplmentary materials, to obtain the dendrite tip temperature (*T_tip_*) and interface velocity (*V*) at a given Péclet number (*Pe*) and temperature gradient (G), for an alloy with the nominal composition (*c*_0_):

(1) $$k_{v}^{i}=\frac{k_{0}^{i}+a_{0}V/D_{i}}{1+a_{0}V/D_{i}}$$

(2) $$m_{v}^{i}=m_{0}^{i}\;\left[\frac{1-k_{v}^{i}\left(1-\ln\;\left(k_{v}^{i}/k_{0}^{i}\right)\right)}{1-k_{0}^{i}}\right]$$

(3) $$c_{liq}^{i,*}=\frac{c_{0}^{i}}{1-\left(1-k_{v}^{i}\right)Iv\left(Pe^{i}\right)}$$

(4) $$Pe^{i}=\frac{vR}{2D_{i}}$$

(5) $$4\pi^{2}\Gamma \left(\frac{1}{R^{2}}\right)+\left(2\sum_{i}\left(m_{v}^{i}Pe^{i}\left(1-k_{v}^{i}\right)c_{liq}^{i,*}\xi_{c}^{i}\right)\right)\;\left(\frac{1}{R}\right)+G=0$$

(6) $$\xi_{C}^{i}=1-\frac{2k_{v}^{i}}{2k_{v}^{i}-1+\sqrt{1+{\left(2\pi /Pe^{i}\right)}^{2}}}$$

(7) $$T_{tip}=T_{liq}+\sum_{i}\left(c_{liq}^{i,*}m_{v}^{i}-c_{0}^{i}m_{0}^{i}\right)-\frac{2\Gamma }{R}-\frac{V}{\mu }-\frac{GD}{V}$$

Here, $k_{v}^{i}$ and $m_{v}^{i}$ are velocity-dependent partition coefficients and liquidus slopes, respectively, calculated using the model by Aziz [27] and the driving force for interface movement during rapid solidification. $k_{0}^{i}$ and $m_{0}^{i}$ are the equilibrium partition coefficients and liquidus slopes, respectively. $c_{liq}^{i,*}$ is the liquid composition at the dendrite tip and *a*_0_ is the characteristic diffusion distance or solute jump distance at the interface. *D_i_* is the solute diffusivity of element *i* in the liquid. Γ, the Gibbs-Thomson coefficient, is calculated as the solid-liquid interfacial energy divided by the entropy of fusion per unit volume of liquid at the liquidus temperature (*T_liq_*). Finally, *μ* is the interface kinetic coefficient.

The model was used to simulate the solidification of the alloy with the primary phase chosen as austenite and *δ*-ferrite, respectively, in separate simulations. The input parameters used for these calculations were *D_i_* = *D* = 5•10^−9^ m^2^/s for all elements, a_0_ = 5•10^−9^ m, and *μ* = 10 m/s/K. For austenite, *T_liq_* for the austenite-liquid equilibrium and Γ were calculated to 1749.3 K and 5.6•10^−8^ m•K, respectively. For *δ*-ferrite, the *T_liq_* for the *δ*-ferrite-liquid equilibrium and Γ were calculated to 1758.4 K and 5.5•10^−8^ m•K, respectively. $k_{0}^{i}$ and $m_{0}^{i}$ were evaluated at every iteration using a material-specific Calphad thermodynamic database.

The high cooling rates during the L-PBF process prevent any diffusional transformation and enable the austenite to transform to martensite when the M_s_ temperature is reached. To investigate the effect of elemental micro-segregation on the M_s_ temperature, the M_s_ temperature model implemented in the Thermo-Calc software was applied. The model is a semi-empirical thermodynamic model based on the work by Borgenstam and Hillert [7] and Stormvinter et al. [8]. The model was used to calculate the M_s_ temperature at each composition along the elemental segregation profile predicted by the DICTRA solidification simulations.

For all calculations described above, the composition Fe-0.35C-4.93Cr-0.45Mn-2.24Mo-0.049N-0.25Si-0.54V (weight-percent, wt.%) was used. The Thermo-Calc Software TCFE Steels/Fe-alloys database version 9 [29] was employed for the M_s_ temperature calculation and for the calculations of the solid-liquid interfacial energies for the tip temperature model. For all other calculations, materials specific Calphad thermodynamic and diffusion mobility databases were applied.

## 4. Results and discussion

### 4.1. Solidification behavior

**Fig. 1.**
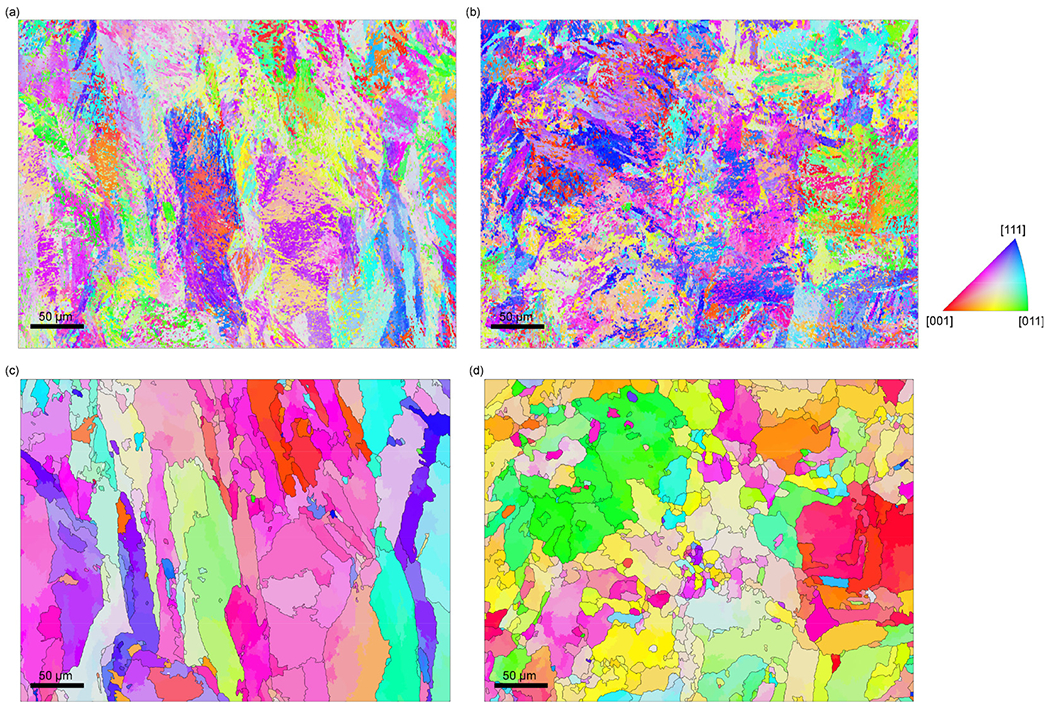
Orientation maps for the martensite (a) parallel and (b) perpendicular to the building direction. Reconstruction of the prior austenite grain structure from EBSD maps of the as-built microstructure (a) parallel to the building direction and (b) perpendicular to the building direction. Columnar prior austenite grains are observed.

The microstructure of the as-built material is formed by solidification followed by transformation of austenite to martensite. In Fig. 1 , prior austenite grains, reconstructed from the EBSD orientation maps of the as-built microstructure (Fig. 1 (a) and (b)), are shown for the microstructure parallel (Fig. 1 (c)) and perpendicular to the building direction (Fig. 1 (d)), respectively. The powder layer thickness during the AM process was 30 μm and most of the prior austenite grains elongated in the building direction (Fig. 1 (c)) thus span over several layers. This suggests that epitaxial growth of austenite grains from the grains of the already solidified material in the underlying layer occurs as is common for the powder-bed fusion based AM techniques [2,22].

**Fig. 2.**
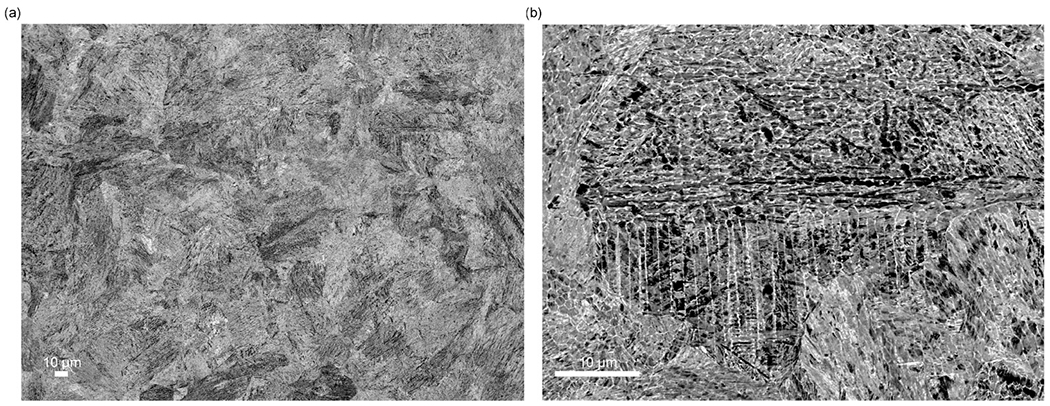
SEM images of the as-built microstructure parallel to the building direction; (a) overview and (b) high magnification showing a martensitic structure and a cellular segregation substructure.

**Fig. 3.**
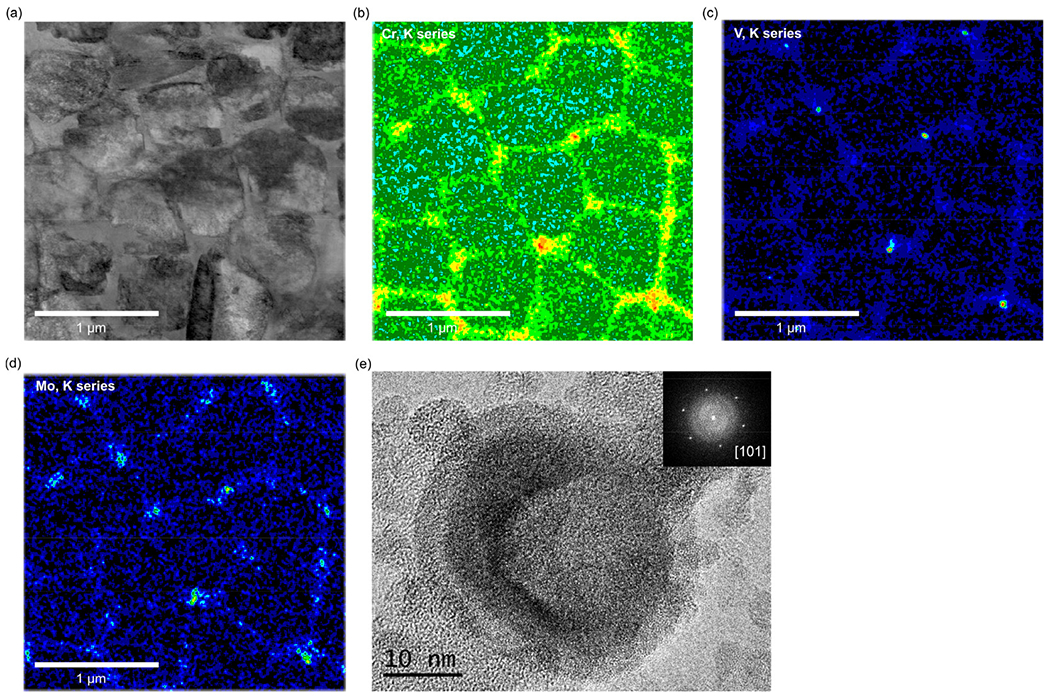
(a) STEM-HAADF image and (b) Cr, (c) V and (d) Mo X-ray intensity maps of the cellular substructure of the as-built material (from a thin foil specimen). (e) HRTEM image of carbides at the cell junctions and corresponding FFT identifying them as hexagonal (P63/mmc) M_2_C (from an extraction replica specimen).

SEM images of the as-built microstructure show a martensitic matrix phase (Fig. 2 (a)) and in higher magnification, a cellular substructure (Fig. 2 (b)). The cell boundaries are enriched in Cr, Mo and V as can be seen in Fig. 3 where a high-angle annular dark-field (HAADF) STEM image and STEM-EDS elemental maps are shown. The HAADF-STEM and the EDS maps also reveal that nano-sized precipitates are located at the cell junctions in the as-built microstructure. These precipitates, identified as hexagonal M_2_ C carbides (see Fig. 2 (e)) using high-resolution TEM (HRTEM) and fast Fourier transform (FFT) analysis, probably form in the last solidified liquid. They are, enriched in Cr, Mo and in particular V which presumably is a result of the local composition in these most segregated regions. The C level in these precipitates could not be verified experimentally.

**Fig. 4.**
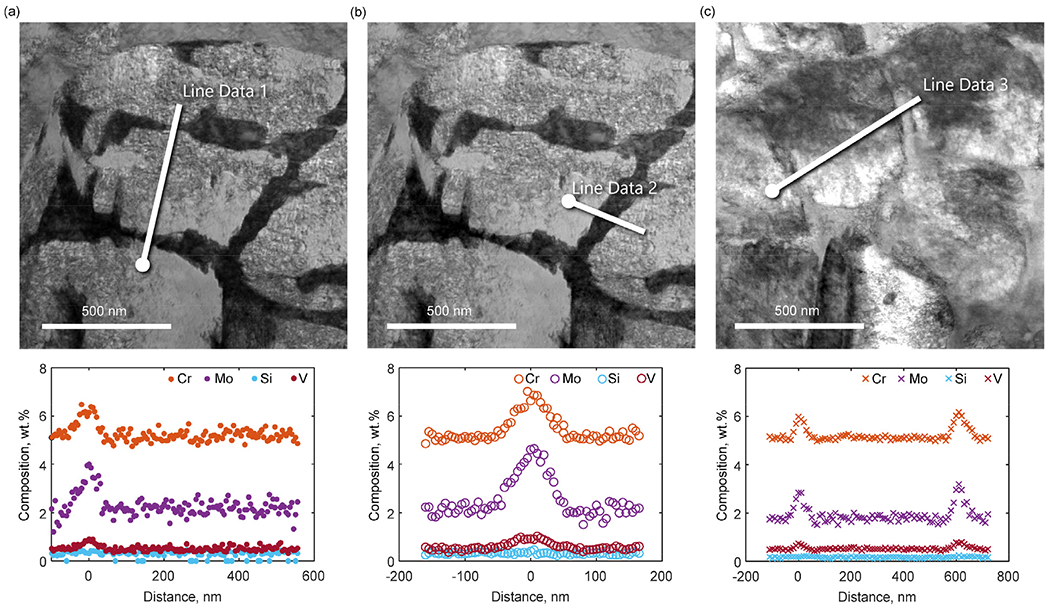
STEM-EDS line scans over inter-cellular boundaries at three different locations in the as-built microstructure.

To quantify the micro-segregation of Mo, V and Cr, STEM-EDS line scans over the inter-cellular regions were performed and in Fig. 4, the results are shown for three different locations. The diameter of the cellular structure is around 500-600 nm and the width of the micro-segregated regions at the cell boundaries and the level of segregation vary depending on the location of the measurements. For locations 1 and 2, the level of segregation is higher compared to location 3. For example, the Mo content in the intercellular region for locations 1 and 2 are over 2 wt.% higher than in the center of the cells. At location 3, the Mo content in the intercellular region is instead only about 1 wt.% higher than in the cell center. This can be explained by the complex thermal evolution during L-PBF where different segregation profiles and cell sizes can be attributed to local variations in the thermal history.

**Fig. 5.**
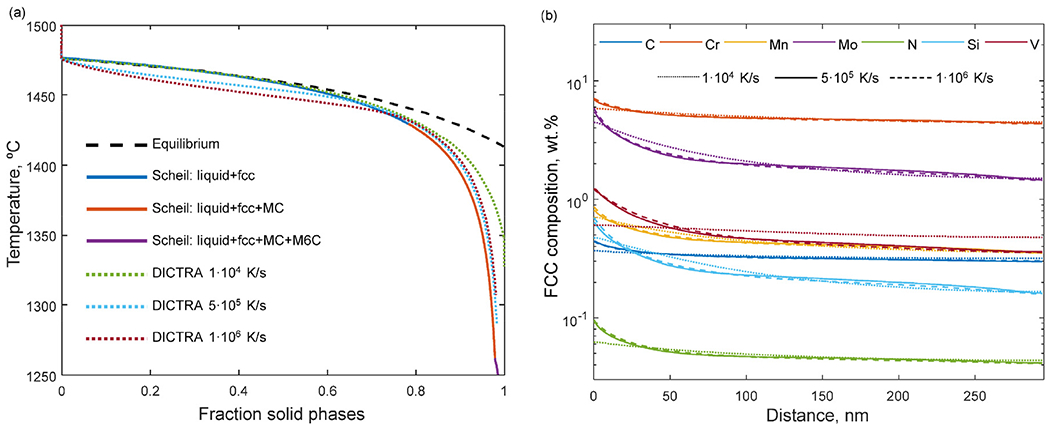
(a) Solidification paths predicted by the Scheil-Gulliver model and DICTRA in comparison to the equilibrium path. (b) Elemental micro-segregation as a function of distance from the inter-cellular boundary when 98% of the system has solidified calculated using DICTRA.

**Fig. 6.**
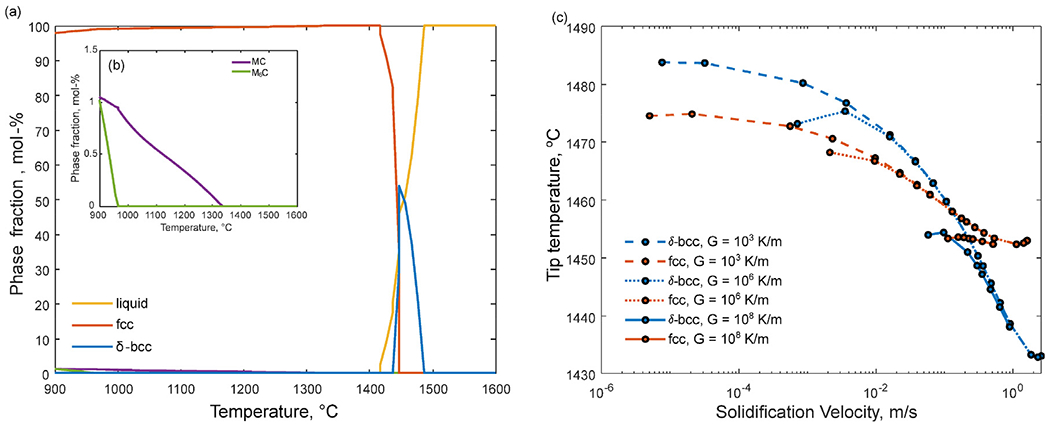
(a)-(b) Calculated equilibrium phase fractions. *δ*-ferrite is stable at high temperatures (a) and MC is the equilibrium carbide at the austenitization temperature of 1010°C (b). (c) Calculated tip temperature as a function of solidification velocity for *δ*-ferrite (*δ*-bcc) and austenite (fcc). At high velocities, a transition is expected from *δ*-ferrite to austenite as the primary solidification phase.

In Fig. 5 (a), the solidification path predicted by the Scheil-Gulliver model with C and N entered as *fast diffusers* is shown along with the solidification paths predicted by DICTRA for different cooling rates. According to equilibrium calculations, *δ*-ferrite is stable at higher temperatures (Fig. 6 (a)) but, as previously described, it has been suspended for both the Scheil-Gulliver and the DICTRA simulations. This is supported by the tip temperature calculations. Fig. 6 (b) shows the calculated tip temperature as a function of solidification velocity for a wide range of thermal gradients (G) including the ranges expected for L-PBF [30,31] which are influenced by many factors such as process parameters, scanning strategy, part geometry and convection [32,33]. At high velocities, austenite indeed has a higher dendrite tip temperature than *δ*-ferrite, and is more likely to form when the alloy solidifies at higher solidification velocities. Due to the model approximations assuming, for example, that steady-state holds and the potential uncertainties associated with the input parameters, exact quantitative agreement with experiments is not expected. However, based on the experimental observations of phases and segregation showing no indications of the presence of *δ*-ferrite in the as-built microstructure, and the trend of these calculations, it is hypothesized that no *δ*-ferrite forms during solidification.

For the Scheil-Gulliver calculation, austenite is thus assumed to be the primary solidification phase and as the alloying elements segregate to the liquid phase, the model predicts formation of MC as well as M_6_ C during the very last stage of the solidification when the most segregated liquid solidifies. This is not in agreement with the experimental observations which suggest that M_2_C forms during the L-PBF process of this steel. This discrepancy could be due to the simplified Scheil-Gulliver model where diffusional effects are ignored and local equilibrium is assumed, or inaccuracies in the thermodynamic database applied for the calculations.

The predictions using the Scheil-Gulliver model and DICTRA lead to similar fraction of solid versus temperature curves (Fig. 5 (a)). The DICTRA solidification paths for the two highest solidification rates, 5•10^5^ K/s and 1•10^6^ K/s, are comparable to the path predicted by the Scheil-Gulliver model whereas the path for the lower solidification rate, 1•10^4^ K/s, lies between the Scheil-Gulliver predictions and the calculation at equilibrium (dashed line). The solid fraction curves of the DICTRA simulations also lay slightly below the Scheil-Gulliver curve for lower fraction of solid and above for higher fraction of solid. This is due to the diffusion in the liquid and solid phases which is fully accounted for in DICTRA but not in the Scheil-Gulliver model. In the DICTRA simulation, alloying elements accumulate at the interface in the liquid creating a compositional gradient during the first stage of solidification. This affects the solid-liquid interface velocity and hence, the solid fraction evolution is different from the Scheil-Gulliver curve where the liquid instead is treated as homogeneous throughout the calculation. At a later stage of solidification, the composition gradient in the liquid phase levels out and the difference between the DICTRA curves and the Scheil-Gulliver curves can be explained by diffusion in the solid phase accounted for in DICTRA.

In Fig. 5 (b), the solid phase composition at the end of solidification, when 98% of the system is solid, is shown as a function of distance, from the inter-cellular region to the center of the cell, for the different cooling rates. All alloying elements, in particular C, Cr, Mo, N and V, segregate towards the inter-cellular region which agrees with the experimental observation. The micro-segregation profiles predicted for the higher cooling rates are comparable whereas the lower cooling rate leads to a slower solidification process with more time for elemental back-diffusion and thus less severe segregation and a wider segregation region.

**Fig. 7.**
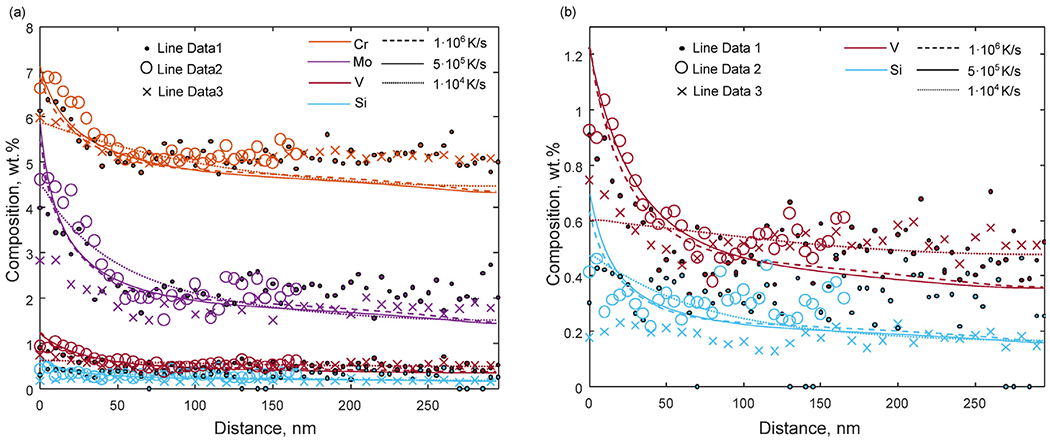
(a) Calculated segregation profile as a function of distance, from the inter-cellular region to the center of the cell, for (a) Cr, Mo, Si and V and (b) Si and V in comparison to the STEM-EDS line scans Line Data 1, 2 and 3.

In Fig. 7 , the calculated segregation profiles are compared with the STEM-EDS line scans for Cr, Mo, Si and V. By comparing the extension of the segregation zone, it can be concluded that the DICTRA calculations with the two highest cooling rates are in closer agreement with the experiments than the result for the lower cooling rate. This suggests that the cooling rates during this L-PBF process are in the range 5•10^5^−1•10^6^ K/s. Although the level of agreement between the DICTRA calculations and the measurements depends on the location of the measurement, the results show that the calculations give a reasonable estimation of the micro-segregation during L-PBF of this material. For the current measurements, the calculated results agree better with the measurements at locations 1 and 2 than with the measurement at location 3. The calculations also provide an indication of the behavior of C and N during the L-PBF processing which would be difficult to measure accurately experimentally. However, the calculations do not include the cyclic heating and cooling in the solid state after solidification when some redistribution of C and N cannot be ruled out depending on the process condition and the scanning strategy.

### 4.2. Retained austenite

**Fig. 8.**
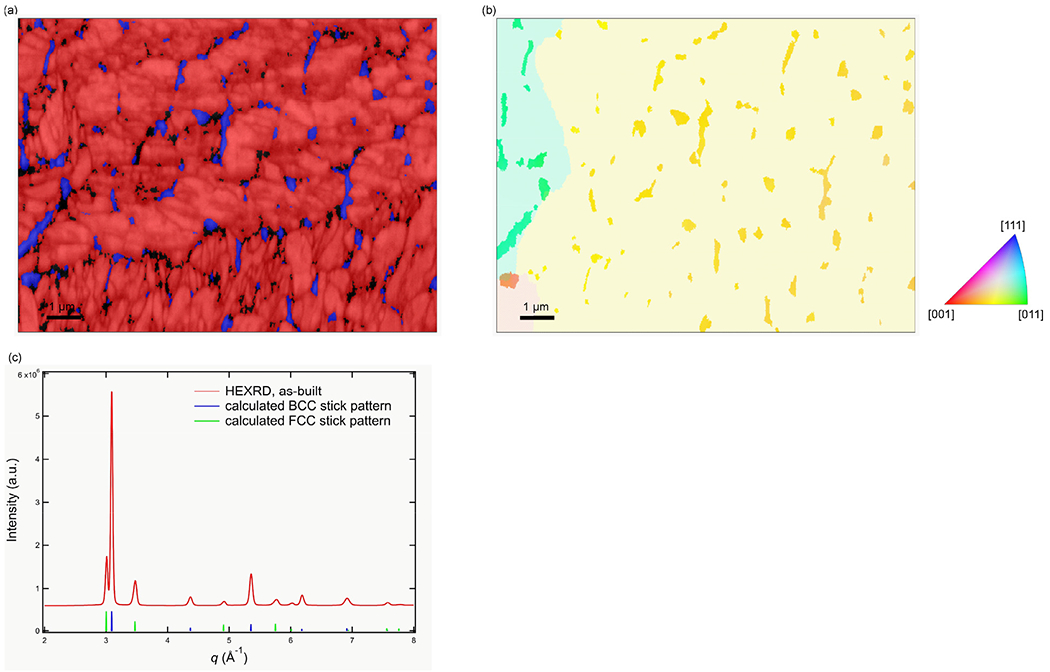
(a) Phase-colored EBSD map showing austenite (blue) and martensite (red), (b) measured austenite orientation shown on the reconstructed prior austenite orientation and (c) HEXRD data showing the bulk phases in the as-built steel

A phase colored EBSD map (Fig. 8 (a)) of the as-built structure shows retained austenite (blue colored) in the segregated regions. In Fig. 8 (b), the measured orientation of the austenite is compared with the reconstructed prior austenite orientation indicating that the observed network of austenite stems from the retained austenite rather than from nucleation and growth of new austenite. The phase fraction of retained austenite is (16.1 ± 0.2)% according to the HEXRD measurements (Fig. 8 (c)).

**Fig. 9.**
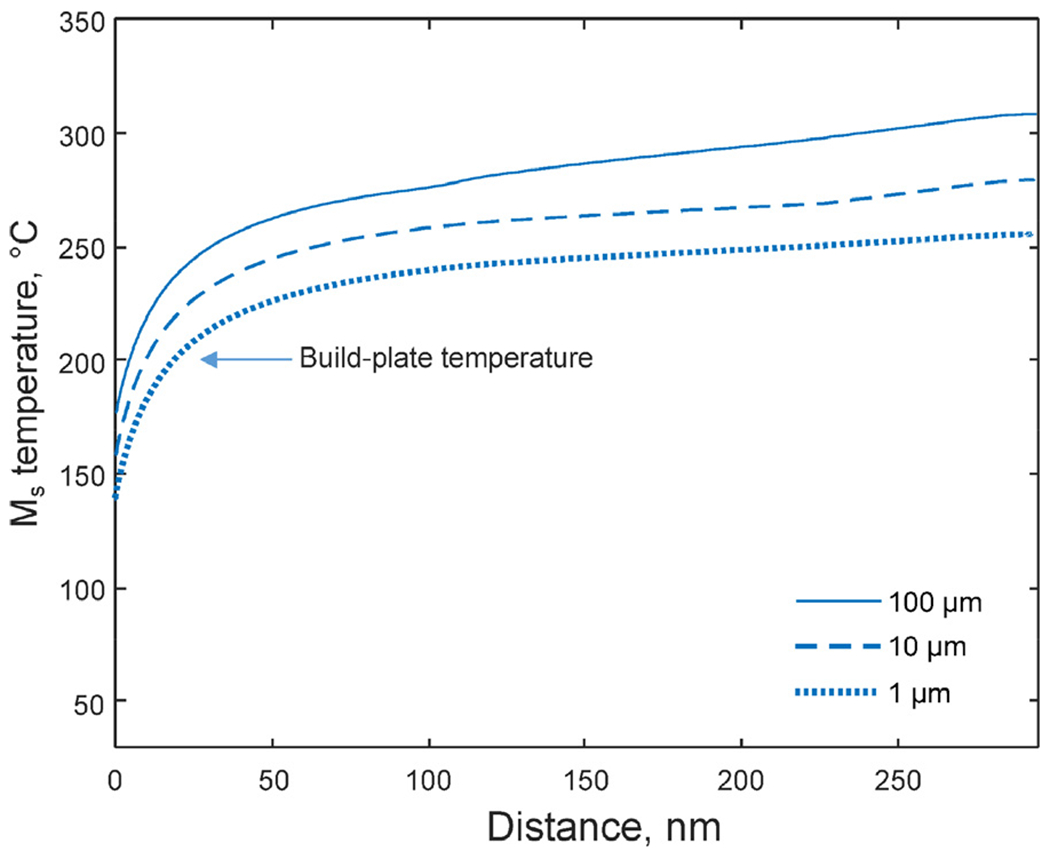
Calculated M_s_ temperature as a function of segregation profile for different austenite grain sizes using the DICTRA results for 5•10^5^ K/s as input. The M_s_ temperature decreases as the inter-cellular region is approached suggesting that the lower M_s_ temperature causes retainment of austenite in these regions.

The presence of the retained austenite in the as-built microstructure can be understood by the spatial composition variation caused by the micro-segregation during the L-PBF process and how it affects the M_s_ temperature. For the present composition (Table 1) and assuming a prior austenite grain size of 100 μm, the M_s_ temperature for this material is predicted to be 266 °C using the M_s_ temperature model. The M_s_ temperature is strongly dependent on alloying, and for the current steel, all alloying elements have a reducing effect on the M_s_ temperature [34]. Hence, the enrichment of alloying elements at the inter-cellular region decreases the M_s_ temperature with respect to its nominal value. This is confirmed in Fig. 9, where the calculated M_s_ temperatures as a function of distance from the inter-cellular region are shown. Here, the composition profile from the DICTRA solidification prediction for the cooling rate 5•10^5^ K/s was used as input. In addition to the chemical influence on the M_s_ temperature, the prior austenite grain size also influences it. Austenite grain sizes below a critical value lead to stronger austenite and to initiate shear transformation for martensite formation, a higher critical driving force and hence, lower M_s_ temperature are required [35]. The prior austenite grains in the current work are elongated with varying lengths and widths (Fig. 1 (c) and (d)). A mean value of the prior austenite grain size can be estimated from the EBSD data by accounting for images of the structure parallel as well as perpendicular to the build direction. However, since the standard deviation is quite large, the M_s_ calculations were performed for a range of grain sizes (1 μm, 10 μm and 100 μm) instead of assuming one specific value to get an estimation of the variation of this effect. The result shows that the M_s_ temperature decreases as the inter-cellular region is approached (Fig. 9), suggesting that lower temperatures need to be reached for the martensite transformation to occur in these regions compared to the centers of the cellular solidification substructure. Potential build-up of dislocations in the segregated intercellular regions prior the austenite to martensite transformation as observed for, e.g., austenitic stainless steel processed by L-PBF [36], may also contribute to the retainment of austenite via mechanical stabilization of the austenite [37]. It is, however, worth noting that the influence of the compositional gradient is rather large, about ~150 ^0^C difference between inter-cellular regions and cell centers (Fig. 9) is predicted. This can be compared with the effect of the austenite grain size which is in the order of ~50 ^0^C for the current calculations.

**Fig. 10.**
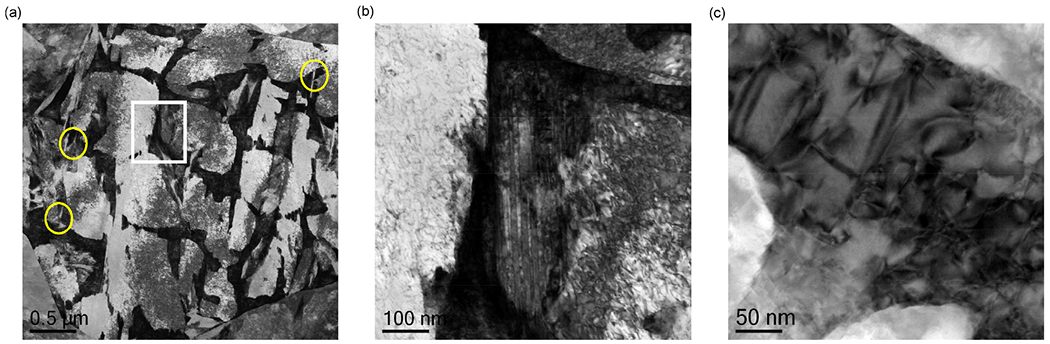
STEM images of the inter-cellular region for different magnifications showing (a) a network of austenite mixed with larger martensite units together with finer martensite units in the austenite network (marked in yellow), (b) a highly twinned martensite adjacent to the austenitic network in higher magnification (inset in (a)) and (c) dislocations in the retained austenite network.

Furthermore, it is also worth noting the distribution of martensite in relation to the network of austenite. From the calculated variation in M_s_ temperature one could expect martensite to form at different stages during cooling. Most of the microstructure has a similar M_s_ temperature but a significantly lower transformation temperature is expected in the segregated regions. The martensite units are relatively large and are not confined within one cell but typically span over an area containing several of the cellular solidification substructure units (Fig. 10 (a)). At some locations in Fig. 10 (a), what appears to be a clearly finer martensite with a different morphology is observed in the austenite network. This suggests that also the segregated austenite can decompose into martensite even if this is not frequently observed. Furthermore, the highly twinned morphology seen in Fig. 10 (b) indicates a lower transformation temperature compared to the adjacent larger martensite units.

The material studied in this work, was processed with a build-plate temperature kept at 200 °C. It is reasonable to assume that even though the temperature history is complex, the temperature in the component never falls much below 200 °C at any point during the process. This implies that in the most segregated, intercellular regions, the M_s_ temperature is not reached and the austenite is retained during the L-PBF processing. The calculations show that the lowest M_s_ temperature is obtained for the smallest grain size and in the most severely segregated regions which is expected since the stability of the austenite increases with decreasing austenite size [38]. For the grain size of 1 μm, the lowest M_s_ temperature is just below 150 °C. This is well above room temperature and implies that the material is cooled down to temperatures below the M_s_ temperature when the L-PBF process is completed and the built-plate is not any longer heated. However, the regions with retained austenite have nevertheless not transformed to martensite. This suggests that, in addition to the compositional influence on the austenite stability and the resulting M_s_ temperature, the austenite in the small-sized inter-cellular regions is mechanically stabilized [39,40] by the residual stresses normally present in L-PBF processed material.

Heating of the build plate is common when processing martensitic tool steels with intermediate carbon content by L-PBF [3–6] to avoid issues with cracking and delamination. The retained austenite present in the steel studied here may therefore play a role in the processability of this steel. In the STEM images (Fig. 4) of the as-built microstructure, the retained austenite regions appear dark due to the high dislocation density. This is shown in higher magnification in Fig. 10 (c) and suggests that these regions are exposed to plastic strain during the process. Hence, the retained austenite could be an important attribute of the evolving microstructure to accommodate the stresses associated with the martensitic transformation and thus prevent cracking. The role of retained austenite for the processability of these types of steels should therefore be studied further while accounting for both L-PBF and post-processing aspects as well as targeted final material properties. Austenite retained in the martensitic matrix will affect the mechanical properties of these kind of steels [41], and for most applications, its presence is not beneficial. For this reason, post heat treatment is necessary.

### 4.3. Austenitization

**Fig. 11.**
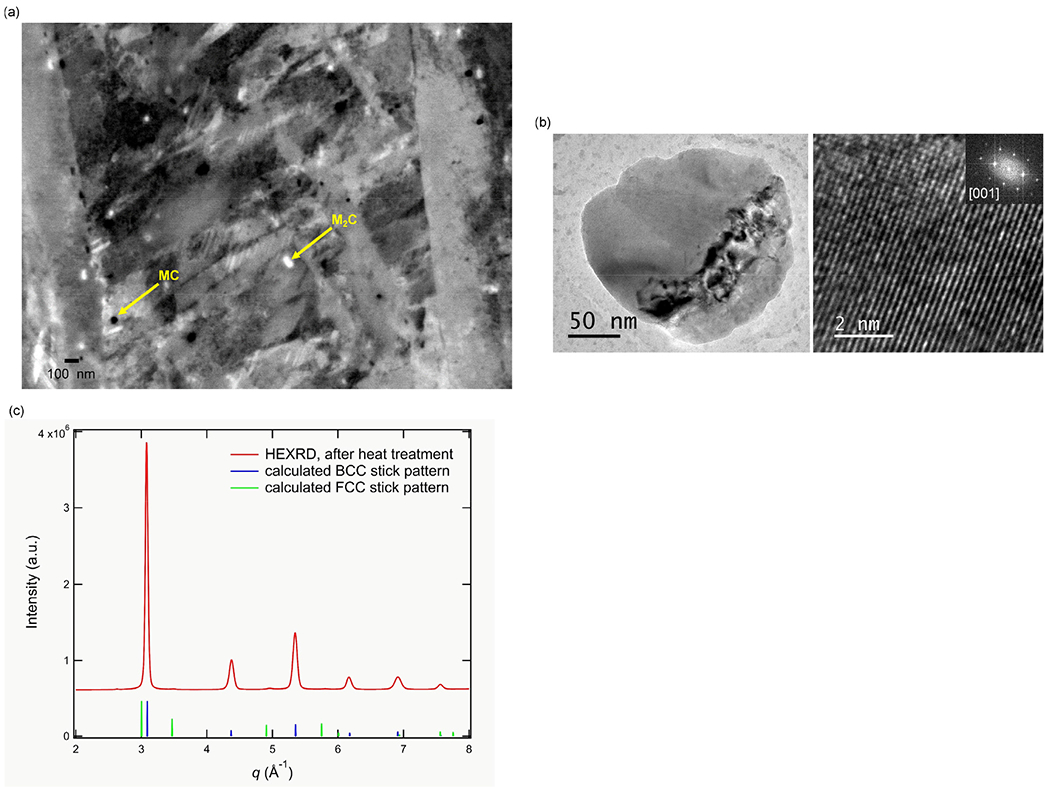
(a) SEM image of the microstructure after austenitization for 1 h at 1010 °C followed by water quenching. Two types of precipitates are observed in the martensite matrix; M_2_ C and MC. (b) TEM and HRTEM image and FFT analysis identifying the halite-type (Fm-3m) MC precipitate and (c) HEXRD data showing that the matrix has been nearly completely transformed to martensite.

A SEM image of the microstructure after heat treatment for 1 h at 1010 °C is shown in Fig. 11 (a). The BE contrast suggests that two carbides with different compositions are present. The composition of the carbides was measured by STEM-EDS (Table 2), and the crystal structures were identified as the hexagonal M_2_C carbide (same type of carbide observed in the as-built condition (Fig. 2 (e)) and the cubic halite-type MC carbide, respectively (Fig. 11 (b)).

**Fig. 12.**
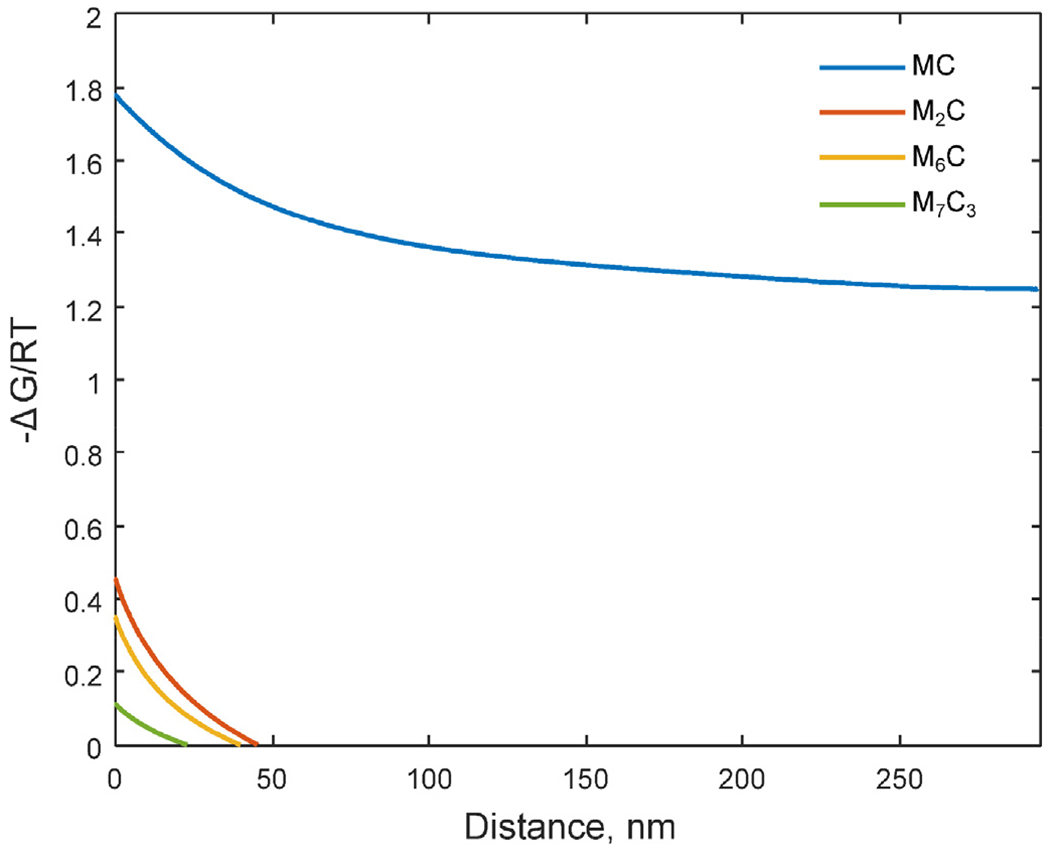
Calculated driving force for precipitation of the carbide phases MC, M_2_C, M_6_C and M_7_C_3_ from an austenitic matrix as a function of distance from the intercellular region. The start composition for the calculation is the DICTRA solidification simulation result for the cooling rate 5•10^5^ K/s.

According to the equilibrium calculation, the stable carbide at the austenitization temperature, 1010 °C, for this steel is only MC (Fig. 6 (b)) and not both MC and M_2_C as observed experimentally. In Fig. 12, the calculated thermodynamic driving forces for formation of the carbides from austenite at 1010 °C, are shown as a function of the segregation profile predicted by the DICTRA solidification simulation (5•10^5^ K/s). According to the calculation, the driving force for MC formation is the highest and formation of MC is thermodynamically preferable over the whole segregation composition profile as expected from the equilibrium calculations at 1010°C for the nominal composition. The calculations also show that, locally in the most segregated area, M_2_C, M_6_C and M_7_C_3_ are thermodynamically favorable to form. In particular, the driving forces for M_2_C and M_6_C formation are enhanced in this region. The M_2_C carbide forms during the L-PBF processing (Fig. 3) and the fact that it is still observed in the microstructure after the austenitization heat treatment at a temperature where it is not stable according to the nominal composition suggests that the locally segregated microstructure does not homogenize fast enough to destabilize the M_2_C carbide and give it time to dissolve. However, the redistribution of C and N is fast enough for them to diffuse to form cubic V-rich carbonitride during the austenitization. To dissolve the M_2_C carbide and release more alloying elements for the secondary precipitation during tempering, a longer austenitization time or a higher austenitization temperature should be applied.

However, the heat treatment and the quench from the austenitization temperature do cause almost all austenite to transform to martensite. This is shown by the HEXRD measurement (Fig. 11 (c)) where a very low austenite fraction, (0.2 ± 0.1)%, is present after austenitization. In that sense, the austenitization treatment of the L-PBF processed material produces a microstructure that resembles the microstructure after austenitization of a conventionally processed tool steel. Thus, the microstructure response to subsequent tempering treatments is expected to be similar for both processes even though the secondary carbide fractions can vary due to the different amounts and types of austenitization carbides.

The microstructure response of the as-built microstructure if tempered directly, on the other hand, will differ from that of the conventionally processed material. The retained austenite will likely transform in connection to the tempering treatment also in the case of direct tempering after L-PBF processing but may lead to a higher amount of newly formed martensite than in the conventional material after tempering. The characteristics of the as-built microstructure with the compositional variation will most likely also affect the precipitation kinetics. This should be further studied in future work.

## 5. Summary and conclusions

In this work, the microstructure of a medium carbon hot-work tool steel processed by L-PBF is studied in detail. Computational thermodynamics and kinetics tools are used to interpret the as-built microstructure and its response to post heat treatments. Using a combination of characterization techniques, quantitative microstructure data is obtained enabling detailed comparisons with the calculated results.

The high solidification and cooling rates during the L-PBF process suppress the *δ*-ferrite formation and the solidification process results in a micro-segregated austenite that partly transforms into martensite at lower temperatures (16% austenite was detected in the as-built microstructure in this case). The high solidification rate produces a cellular solidification substructure where the alloying elements have segregated to the inter-cellular regions and where metastable M_2_C solidification carbides have formed in the cell junctions. This spatial composition variation, in turn, lowers the M_s_ temperature in the inter-cellular regions where retained austenite is present after the L-PBF processing.

By reducing the complex solidification behavior during AM to a diffusion problem in one dimension, the micro-segregation during solidification is calculated and enables detailed comparisons with the measured segregation profiles quantified at a nanometer scale. The calculated solidification results are used as input for further computational analysis including prediction of the M_s_ temperature variation. The result shows that the calculations provide a reasonable estimation of the micro-segregation during L-PBF and explains the location and amount of retained austenite observed experimentally. This demonstrates the power of computational tools based on computational thermodynamics and kinetics for designing alloys for AM by reasonable predictions of the alloys’ response to the L-PBF process and post heat treatments. Accounting for rapid solidification effects and region-specific thermal history, the predictive capability of these tools can be improved. Moving forward, quantitative predictions of micro-segregation and phase transformations can be integrated into component-level design in the industry, strengthening the links of process-structure-property based model predictions.

## Supplementary Material

Supp1

## Figures and Tables

**Table 1:** Chemical composition of the feedstock powder in weight-percent (wt.%).

**C**	**Cr**	**Mo**	**V**	**Mn**	**Si**	**N**	**O**	**P**
0.35	4.93	2.24	0.54	0.45	0.25	0.049	0.02	0.007
**S**	**Ni**	**W**	**Co**	**Sn**	**Al**	**Cu**	**Fe**	
0.002	0.055	0.008	0.019	0.003	0.012	0.035	Balance	

**Table 2:** Metallic constituents of the carbides in the austenitized condition measured by STEM-EDS in wt.% (95% confidence) from carbon extraction replica sample.

Phase	V	Cr	Fe	Mo
MC	76.3 ± 2.7	8.5 ± 0.4	1.6 ± 1.0	13.6 ± 2.4
M_2_C	38.6 ± 4.5	11.2 ± 0.6	4.3 ± 1.2	45.9 ± 4.9

## Footnotes

1 Certain commercial equipment, instruments, software or materials are identified in this paper to foster understanding. Such identification does not imply recommendation or endorsement by the Department of Commerce or the National Institute of Standards and Technology, nor does it imply that the materials or equipment identified are necessarily the best available for the purpose.
