# Supplementary material for: Influence of solidification structure on austenite to martensite transformation in additively manufactured hot-work tool steels
Source: Acta Mater. Author manuscript; Available in PMC 2023 Apr 11. (PMC10088486; doi:10.1016/j.actamat.2021.117044)

Appendix

Schematic of the approach to solve interface response function equations through numerical iterations [24].

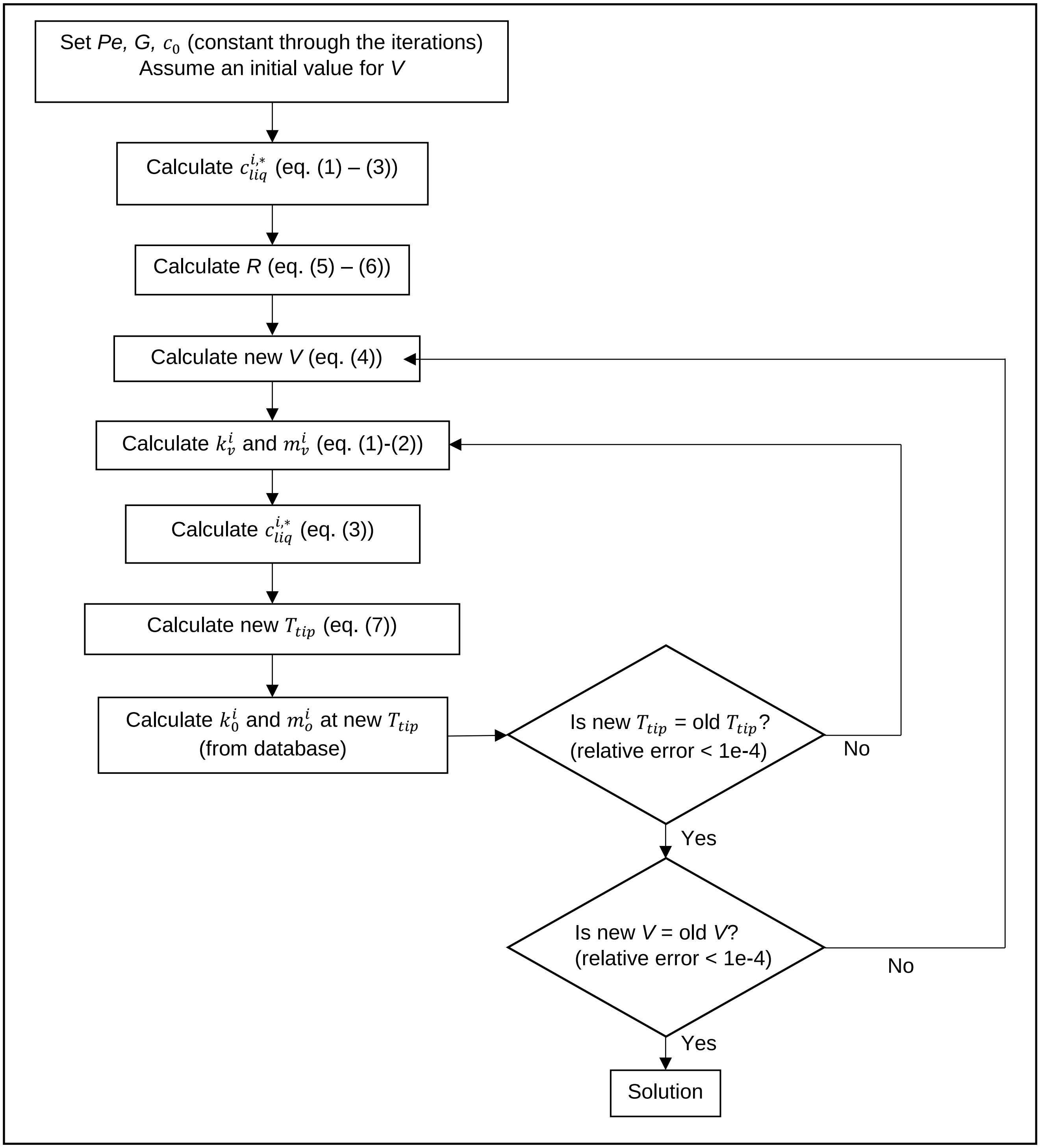

Supplement: Supp1 [file NIHMS1873166-supplement-Supp1.pdf]
